# Extremely Low Frequency Magnetic Field (50 Hz, 0.5 mT) Reduces Oxidative Stress in the Brain of Gerbils Submitted to Global Cerebral Ischemia

**DOI:** 10.1371/journal.pone.0088921

**Published:** 2014-02-19

**Authors:** Snežana Rauš Balind, Vesna Selaković, Lidija Radenović, Zlatko Prolić, Branka Janać

**Affiliations:** 1 Institute for Biological Research, University of Belgrade, Belgrade, Serbia; 2 Institute for Medical Research, Military Medical Academy, Belgrade, Serbia; 3 Department of Physiology and Biochemistry, Faculty of Biology, University of Belgrade, Belgrade, Serbia; National University of Singapore, Singapore

## Abstract

Magnetic field as ecological factor has influence on all living beings. The aim of this study was to determine if extremely low frequency magnetic field (ELF-MF, 50 Hz, 0.5 mT) affects oxidative stress in the brain of gerbils submitted to 10-min global cerebral ischemia. After occlusion of both carotid arteries, 3-month-old gerbils were continuously exposed to ELF-MF for 7 days. Nitric oxide and superoxide anion production, superoxide dismutase activity and index of lipid peroxidation were examined in the forebrain cortex, striatum and hippocampus on the 7^th^ (immediate effect of ELF-MF) and 14^th^ day after reperfusion (delayed effect of ELF-MF). Ischemia *per se* increased oxidative stress in the brain on the 7^th^ and 14^th^ day after reperfusion. ELF-MF also increased oxidative stress, but to a greater extent than ischemia, only immediately after cessation of exposure. Ischemic gerbils exposed to ELF-MF had increased oxidative stress parameters on the 7^th^ day after reperfusion, but to a lesser extent than ischemic or ELF-MF-exposed animals. On the 14^th^ day after reperfusion, oxidative stress parameters in the brain of these gerbils were mostly at the control levels. Applied ELF-MF decreases oxidative stress induced by global cerebral ischemia and thereby reduces possible negative consequences which free radical species could have in the brain. The results presented here indicate a beneficial effect of ELF-MF (50 Hz, 0.5 mT) in the model of global cerebral ischemia.

## Introduction

Cerebral ischemia as a consequence of restricted blood flow, implicating insufficient glucose and oxygen supply, leads to increased production of free radical species [1]. Enormous production of reactive oxygen and nitrogen species (ROS and RNS, respectively) has deleterious effects during pathogenesis of ischemic insult [2], [3]. Brain is highly susceptible to the presence of free radicals due to high content of lipids and relatively low level of endogenous antioxidants [4]. Massive production of ROS might has overall effects on all physiological functions important for surviving. During cerebral ischemia, production of free radicals overwhelm possibility of detoxification and capacity for its removal by enzymes of antioxidative protection like superoxide dismutase (SOD), catalase (CAT), glutathione peroxidase (GPx) and nonenzymatic antioxidants (vitamin C and E, glutathione) resulting in fast and severe damage of cellular proteins, lipids and DNA [3], [5].

Although production of ROS in mitochondria from molecular oxygen presents normal physiological reaction, enormous activation of N-methyl-D-aspartate (NMDA) receptors during cerebral ischemia results in higher production of ROS and nitric oxide (NO). Oxidation of xanthine to hypoxanthine is accompanied by production of superoxide anion (O_2_^−^) and hydrogen peroxide (H_2_O_2_), which further compromises neuronal damage during reperfusion [6], [7]. Peroxidation of lipid membranes produces toxic aldehydes like 4-hydroxynonenal (4-HNE) which damage ion channels, transporters and cytoskeletal proteins. Activation of phospholipase A2 after cerebral ischemia releases arachidonic acid, important source of ROS [8], from membrane phospholipids. Free radicals also activate specific signal pathways like mitogen-activated protein kinase which further contribute to ischemic damage [9].

Production of NO and oxidative stress are also linked to overactivation of poly(ADP-ribose)polymerase-1 (PARP-1), DNA reparating enzyme. PARP-1 overactivation decreases cellular NAD^+^, disturbing NAD^+^-dependent processes like anaerobic glycolysis and mitochondrial respiration, which further induces reduction of ATP content, lack of energy and cell death [10]. Cells of nervous system, astrocytes and microglia, also contribute to level of ROS in cerebral ischemia [11], [12].

One ecological factor whose influence is growing every day due to technological development is extremely low frequency magnetic field (ELF-MF). It has role in the production of free radical species, as well as modulation of antioxidant defense components [13]–[18]. As a omnipresent factor, we can not exclude the impact of ELF-MF on recovery after ischemic insult with possibility of its beneficial effects. In this study we applied ELF-MF (50 Hz, 0.5 mT) for 7 days in gerbils submitted to 10-min global cerebral ischemia and measured oxidative stress parameters in distinct brain structures (forebrain cortex, striatum and hippocampus) on the 7^th^ (immediate effect of ELF-MF) and 14^th^ day after reperfusion (delayed effect of ELF-MF). These results are part of our comprehensive investigations concerning the effects of ELF-MF in animals with experimentally induced cerebral ischemia [19], [20] and contribute to the explanation of spatial and temporal patterns of oxidative stress in the brain of these animals.

## Materials and Methods

### Animals

3-month-old male gerbils (*Meriones unguiculatus*, 55–65 g body weight), obtained from the vivarium of the Institute for Medical Research, MMA, Serbia, were used. Groups of four gerbils per cage (Ehret GmbH & Co. KG, Germany) were placed in an air-conditioned room, at a temperature of 23±2°C, with 55±10% humidity and with lights on 12 h/day (07∶00–19∶00). Commercial food and tap water were given to the gerbils *ad libitum*. All animal procedures were complied with the European Communities Council Directive (86/609/EEC) and were approved by the Ethical Committee for the Use of Laboratory Animals of the Institute for Biological Research, University of Belgrade (Permit Number: 20/08 and 53/10).

### Occlusion of Common Carotid Arteries

Mature gerbils are good model for inducing global cerebral ischemia due to incomplete circle of Willis (lack of collateral communication between the carotid and vertebrobasilar circulation, [21]), so we have done occlusion of both common carotid arteries. A detailed description of the procedure is given in Rauš et al. [19].

### System and Procedure for ELF-MF Exposure

As the source of the alternating MF was used an electromagnet whose detailed characteristics are given in Rauš et al. [19]. It was placed in an isolated room with the same temperature, humidity, light intensity and cycle like in the vivarium. The gerbils were exposed to ELF-MF (50 Hz, 0.5 mT) for 7 days and after that were returned to the vivarium. During experiment, geomagnetic activity was characterized as “very quiet” (Department of Geomagnetism and Aeronomy, Sector for Geodetic Works, Republic Geodetic Authority, Republic of Serbia) measured by a GSM-19 v6.0 proton magnetometer (GEM SYSTEMS INC, Ontario, Canada).

### Experimental Groups

All experiments were performed in a blinded manner. The gerbils were randomly divided into the following groups: Intact, Sham-operated, Sham-exposed, ELF-MF, Ischemia and Ischemia+ELF-MF.

Intact gerbils were not subjected to any type of surgical procedure and/or exposure, and they were the whole time in the vivarium (n = 6). These animals were included in the study to exclude any possibility that the presence of animals in the vicinity of the electromagnet, previously turned off, and/or mechanical stress caused by surgical intervention could have the impact on the measured oxidative stress parameters.

ELF-MF group of gerbils was continuously exposed to the MF (50 Hz, 0.5 mT) for 7 days (n = 13), while Sham-exposed one (n = 6) was submitted to the same experimental procedure as ELF-MF-exposed gerbils with the electromagnet turned off.

Ischemic gerbils were submitted to the 10-min occlusion of both common carotid arteries without (Ischemia, n = 12) or with (Ischemia+ELF-MF, n = 13) exposure to ELF-MF (50 Hz, 0.5 mT) for 7 days. Sham-operated gerbils (n = 6) were submitted to the same surgical procedure as ischemic gerbils, but without occlusion of both common carotid arteries.

The gerbils from Sham-operated, Sham-exposed, ELF-MF, Ischemia and Ischemia+ELF-MF group were further subdivided into two groups. Biochemical analyses were performed on the 7^th^ and 14^th^ day from the beginning of experimental procedure.

### Preparing Tissue for Biochemical Analysis

The gerbils were decapitated and the brains were immediately removed. Forebrain cortex, striata and hippocampus of individual animals were quickly isolated and homogenized in ice-cold buffer containing 0.25 M sucrose, 0.1 mM EDTA, 50 mM K-Na phosphate buffer, pH 7.2. Homogenates were centrifuged twice at 1580 *g* for 15 min at 4°C. The supernatant (crude mitochondrial fraction) obtained by this procedure was then frozen and stored at −70°C. Chemicals were purchased from Sigma (St. Louis, MO, USA). Other chemicals were of analytical grade. All drug solutions were prepared on the day of experiment.

### Nitrite Measurement

NO production was quantified by measuring nitrite, a stable oxidation end product of NO metabolism, by Griess’ method [22]. Briefly, nitrite production was determined by mixing 50 µL of the assay buffer with 50 µL of Griess reagent (1.5% sulfanilamide in 1 M HCl plus 0.15% N-(1-naphthyl)ethylenediamine dihydrochloride in distilled water, v:v). After 10 min of incubation at room temperature, the absorbance at 540 nm was determined and nitrite concentrations were calculated from sodium nitrite (Sigma) standard curve. All measurements were performed in triplicate.

### Superoxide (O_2_^−^) Production and Measurement

In these experiments, O_2_^−^ was measured by the reduction of nitro blue tetrazolium (NBT), as previously described [23]. Detection of this product was by spectrophotometric quantification of a colored formazan product formed from blue tetrazolium. Reduction of NBT was measured at 560 nm. All measurements were performed in triplicate.

### Superoxide Dismutase (SOD) Assay

Total SOD activity, which includes the activity of two SOD isoforms – SOD1 (Cu,ZnSOD) citoplasmatic and SOD2 (MnSOD) mitochondrial isoforms, was measured by the adrenaline method [24]. Inhibition of epinephrine spontaneous auto-oxidation, monitored at 480 nm, was the measure of SOD activity (EC 1.15.1.1). The kinetics of enzyme activity was followed in a sodium carbonate buffer (50 mM, pH 10.2), containing EDTA (0.1 mM), after the addition of epinephrine (10 mM). Enzymatic activity was expressed in units (the amount of sample that causes 50% inhibition of spontaneous epinephrine auto-oxidation) per milligram of protein. All measurements were performed in triplicate.

### Index of Lipid Peroxidation (ILP) Measurement

Malondialdehyde (MDA), the product of polyunsaturated free fatty acids, reacts with thiobarbituric acid, and it is a common ILP. It was measured spectrophotometrically as thiobarbituric acid reactive species. The content of thiobarbituric acid reactive substances formed spontaneously and *in vitro*, stimulated by 0.01 mM Fe^2+^ salts and 0.5 mM ascorbic acid, was measured upon treating the samples with 1 mL of cold thiobarbituric acid-reagent (15% trichloroacetic acid, 0.1 M HC1, 0.75% thiobarbituric acid), and subsequent heating at 95°C in the presence of 50 µM deferoxamine to prevent further iron-catalyzed lipid peroxidation [25]. The absorbance was measured at 533 nm. Control values (without stimulation with Fe^2+^ and ascorbic acid) were determined for each sample. All measurements were performed in triplicate.

### Protein Measurement

The content of protein in the rat brain homogenates (forebrain cortex, striatum and hippocampus) was measured by the method of Lowry et al. [26] using bovine serum albumin (Sigma) as standard. All measurements were performed in triplicate.

### Data Presentation and Statistical Analysis

Data were expressed as means ± SEM (n = 6–8 animals per group). Before statistical analysis, normal distribution of data was assessed using Kolmogorov–Smirnov test. The statistical significance of differences between groups was assessed by one-way analysis of variance. When appropriate, subsequent statistical comparisons were performed by Least Significant Difference (LSD) test.

## Results

There were no differences in values of measured parameters in all examined structures in Intact, Sham-operated and Sham-exposed gerbils (data not shown). Thus, these gerbils were considered as the only Control group (n = 8).

Upon one-way analysis of variance, it was obvious that exposure to ELF-MF significantly affects production of NO and O_2_^−^, SOD activity and ILP in the brain (forebrain cortex, striatum and hippocampus) of gerbils submitted to 10-min global cerebral ischemia (Table 1).

**Table 1:** One-way analysis of variance.

		Forebrain cortex			Striatum			Hippocampus		
	df	F	P	η_p_^2^	F	p	η_p_^2^	F	p	η_p_^2^
NO	6	128.45	<0.001	0.95	37.38	<0.001	0.85	53.58	<0.001	0.89
O_2_^−^	6	63.61	<0.001	0.91	24.00	<0.001	0.79	20.75	<0.001	0.76
ILP	6	20.50	<0.001	0.76	24.49	<0.001	0.79	27.17	<0.001	0.81
SOD	6	50.80	<0.001	0.87	44.56	<0.001	0.87	30.05	<0.001	0.82

df – degrees of freedom; η_p_^2^ – partial eta-squared.

**Figure 1:**
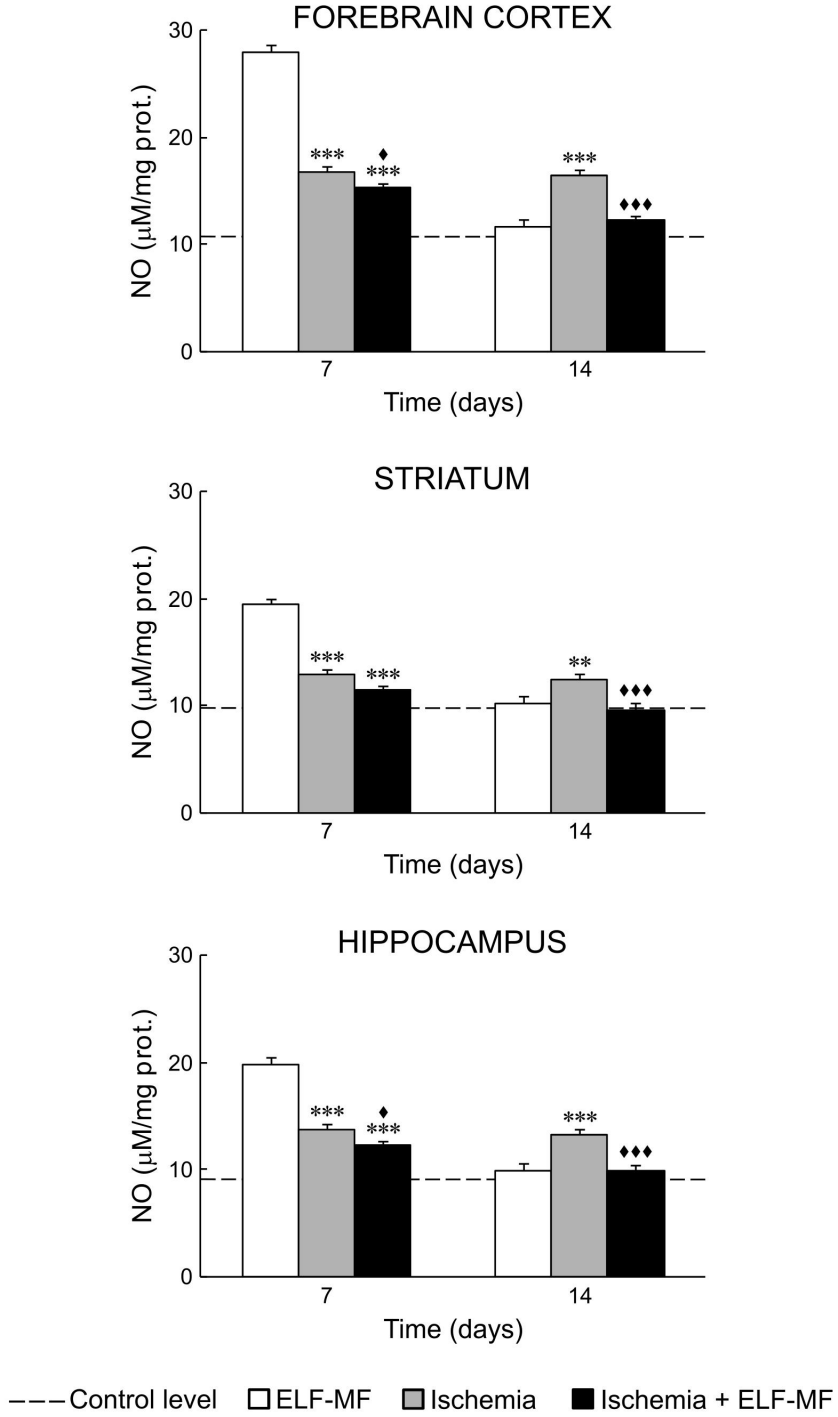
ELF-MF effect on NO content in the brain of gerbils submitted to global cerebral ischemia. Each bar represents mean ± SEM (n = 6–8 animals per group). **p<0.01 and ***p<0.001 indicate significant differences compared to ELF-MF; ^♦^p<0.05 and ^♦♦♦^p<0.001 indicate significant differences compared to Ischemia (one-way analysis of variance followed by LSD test).

**Figure 2:**
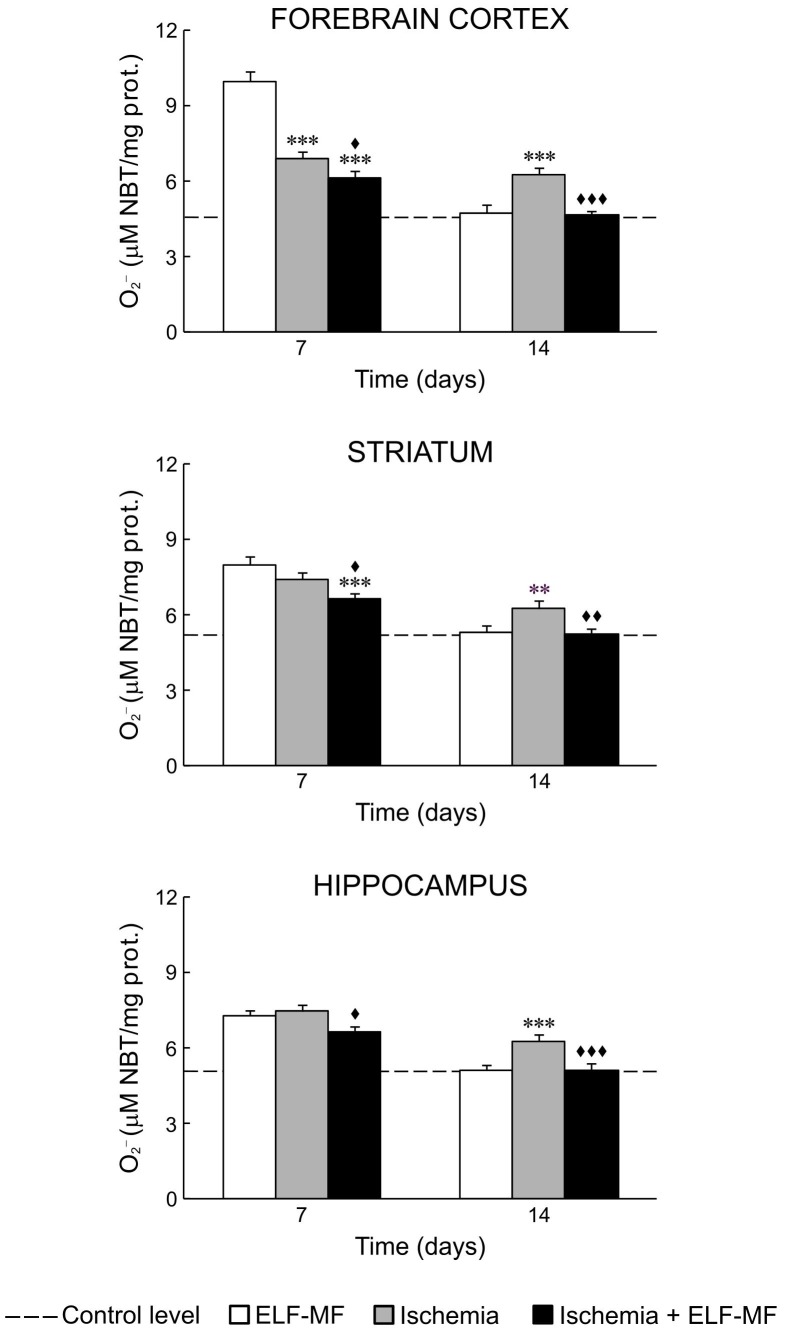
ELF-MF effect on O_2_^−^ content in the brain of gerbils submitted to global cerebral ischemia. Each bar represents mean ± SEM (n = 6–8 animals per group). **p<0.01 and ***p<0.001 indicate significant differences compared to ELF-MF; ^♦^p<0.05, ^♦♦^p<0.01 and ^♦♦♦^p<0.001 indicate significant differences compared to Ischemia (one-way analysis of variance followed by LSD test).

**Figure 3:**
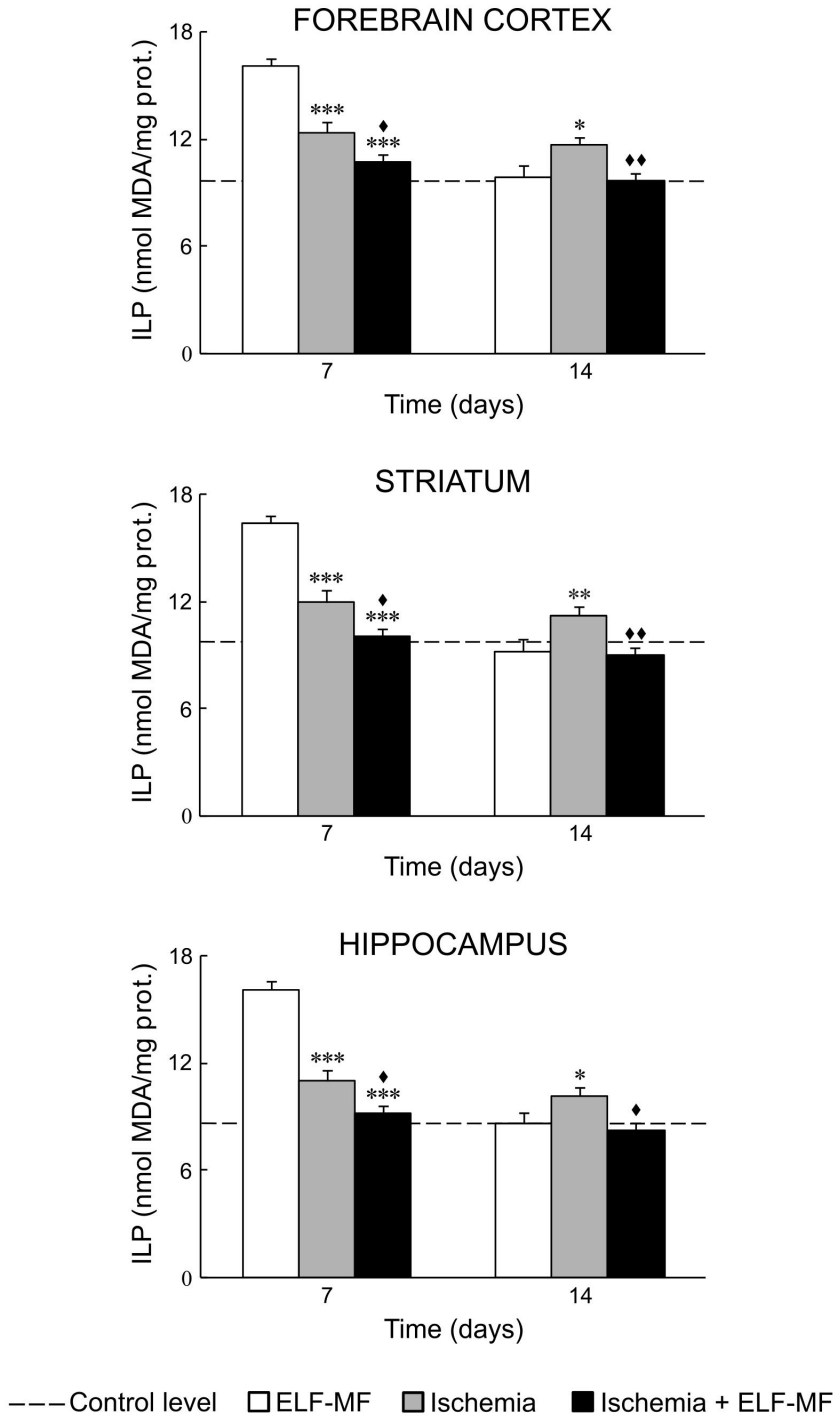
ELF-MF effect on ILP in the brain of gerbils submitted to global cerebral ischemia. Each bar represents mean ± SEM (n = 6–8 animals per group). *p<0.05, **p<0.01 and ***p<0.001 indicate significant differences compared to ELF-MF; ^♦^p<0.05 and ^♦♦^p<0.01 indicate significant differences compared to Ischemia (one-way analysis of variance followed by LSD test).

**Figure 4:**
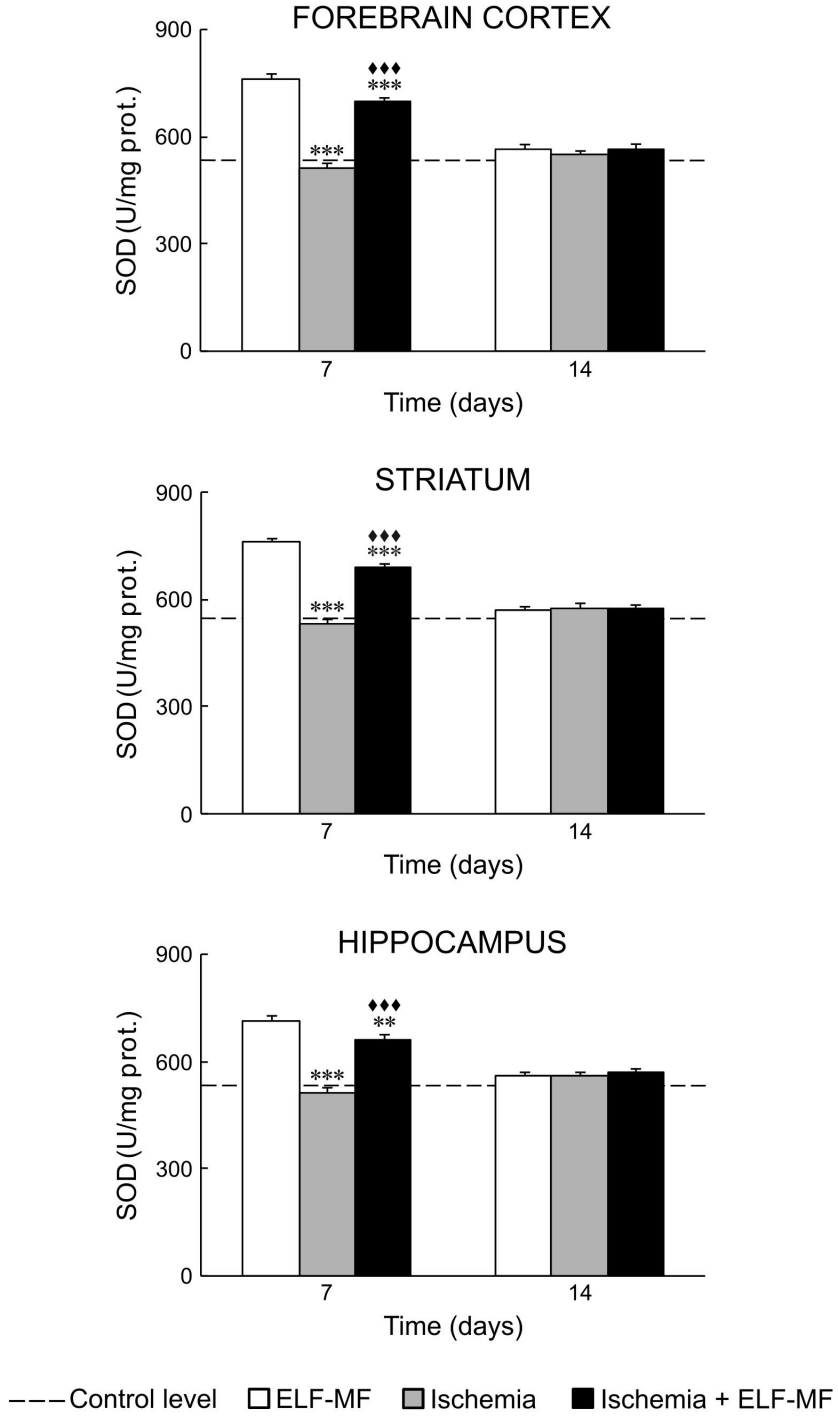
ELF-MF effect on SOD activity in the brain of gerbils submitted to global cerebral ischemia. Each bar represents mean ± SEM (n = 6–8 animals per group). **p<0.01 and ***p<0.001 indicate significant differences compared to ELF-MF; ^♦♦♦^p<0.001 indicates significant differences compared to Ischemia (one-way analysis of variance followed by LSD test).

Our results showed that ischemia *per se* increased oxidative stress in all examined brain structures. It could be seen through increased values of NO, O_2_^−^ and ILP on the 7^th^ and 14^th^ day after reperfusion (Figs. 1, 2 and 3; Tables 2 and 3). SOD activity in these animals was at the control level (Fig. 4; Table 2).

**Table 2:** Significant differences in oxidative stress in the brain of 3-month-old gerbils submitted to 10-min global cerebral ischemia and continuously exposed to ELF-MF (50 Hz, 0.5 mT) for 7 days.

			IMMEDIATE EFFECT			DELAYED EFFECT		
			Cx	S	Hipp	Cx	S	Hipp
		ELF-MF	***	***	***			
NO	Control *vs*.	Ischemia	***	***	***	***	***	***
		Ischemia+ELF-MF	***	*	***	*		
		ELF-MF	***	***	***			
O_2_^−^	Control *vs*.	Ischemia	***	***	***	***	***	***
		Ischemia+ELF-MF	***	***	***			
		ELF-MF	***	***	***			
ILP	Control *vs*.	Ischemia	***	**	***	**	*	*
		Ischemia+ELF-MF						
		ELF-MF	***	***	***			
SOD	Control *vs*.	Ischemia						
		Ischemia+ELF-MF	***	***	***	*		*

Measures are performed on the 7^th^ (immediate effect of ELF-MF) and 14^th^ day after reperfusion (delayed effect of ELF-MF).

Cx – Forebrain cortex; S – Striatum; Hipp – Hippocampus.

*p<0.05, **p<0.01 and ***p<0.001 (one-way analysis of variance followed by LSD test).

**Table 3:** Significant differences (immediate *vs.* delayed effect of ELF-MF) in oxidative stress in the brain of 3-month-old gerbils submitted to 10-min global cerebral ischemia and continuously exposed to ELF-MF (50 Hz, 0.5 mT) for 7 days.

		Cx	S	Hipp
	ELF-MF	***	***	***
NO	Ischemia			
	Ischemia+ELF-MF	***	*	**
	ELF-MF	***	***	***
O_2_^−^	Ischemia		***	**
	Ischemia+ELF-MF	***	***	***
	ELF-MF	***	***	***
ILP	Ischemia			
	Ischemia+ELF-MF			
	ELF-MF	***	***	***
SOD	Ischemia	*	*	*
	Ischemia+ELF-MF	***	***	***

Cx – Forebrain cortex; S – Striatum; Hipp – Hippocampus.

*p<0.05, **p<0.01 and ***p<0.001 (one-way analysis of variance followed by LSD test).

ELF-MF also increased production of free radical species and ILP in the forebrain cortex, striatum and hippocampus on the 7^th^ day (immediate effect of ELF-MF). This increase was to a greater extent than those observed in ischemia (Figs. 1, 2 and 3; Tables 2 and 3). In contrast to ischemia, 7-day exposure to ELF-MF increased SOD activity (Fig. 4; Tables 2 and 3). In this experimental group, 7 days after cessation of exposure (delayed effect of ELF-MF) measured values of oxidative stress parameters were at the control levels (Figs. 1–4; Table 2).

Ischemic gerbils exposed to ELF-MF had also increased values of measured oxidative stress parameters on the 7^th^ day after reperfusion (immediate effect of ELF-MF), but to a lesser extent than animals with global cerebral ischemia or exposed to ELF-MF (Figs. 1–4; Tables 2 and 3). On the 14^th^ day after reperfusion, oxidative stress in the brain of these animals was mostly at the control level (Figs. 1–4; Table 2).

## Discussion

Based on reported results, it is obvious that 7-day exposure to ELF-MF (50 Hz, 0.5 mT) can reduce oxidative stress in the brain of gerbils submitted to 10-min global cerebral ischemia. This effect is the most evident 7 days after cessation of exposure when, in contrast to ischemia, measured parameters were mostly at the control level.

As already described in many papers [1], [27]–[31], cerebral ischemia, due to lack of oxygen and substrate for aerobic metabolism, is accompanied by high production of free radical species in the brain. Our results confirmed once again that cerebral ischemia increases oxidative stress in the forebrain cortex, striatum and hippocampus being almost at the same level on the 7^th^ and 14^th^ day after reperfusion. Free radicals are highly reactive molecules which can disrupt neuronal membranes attacking lipids in molecular bilayer or damaging protein structure, and thus changing its activity and forming protein aggregation [32]. By-product of lipid peroxidation is 4-HNE, toxic aldehyde which damages ion channels, transporters and proteins of cytoskeleton [33]. Cerebral ischemia also activates phospholipase A2 leading to release of arachidonic acid from membrane phospholipid as a new, additional source of ROS [8]. Because of relatively low content of antioxidants and massive production of ROS, cells in ischemic brain are pushed toward death pathways [4].

ELF-MF is unavoidable environmental factor which affects all organisms and recently has application in medicine. Its influence on ionic currents and pumps [34]–[36], neurotransmission [37]–[43] and behaviour [42], [44]–[52] has been well documented. This influence could be achieved through interaction of ELF-MF with chemical bonds between adjacent atoms leading to change in reaction between biomolecules [53] and disruption of biomembrane changing structure of its protein molecules [54]. Based, among others, on this mechanism(s), ELF-MF activates free radical species and prolongs their life [55]–[58].

In our experiment, ELF-MF increased production of NO in all examined brain structures on the 7^th^ exposure day with returning to control level 7 days after cessation of exposure. This increase is in line with previous findings [16], [55], [59], [60]. Activity of NO synthase is mediated through increase of intracellular Ca^2+^, event that occurs as a consequence of the applied ELF-MF [34], [61]–[63]. In case when we exposed ischemic gerbils to ELF-MF, NO content was slightly lower then in only ischemic gerbils on the 7^th^ day after reperfusion, and at the control level on the 14^th^ day after reperfusion. Having in mind that cerebral ischemia also increases influx of Ca^2+^ [64], someone could expect that the effect of ELF-MF would be cumulative leading to additional increase of NO content.

The same results are observed with ILP meaning that ELF-MF could attenuate harmful effects of ischemia on membranes and reduce further ROS and RNS production. Like in our case, in the majority of experiments ELF-MF increases lipid peroxidation [16], [53], [60], [65]. We can propose that ELF-MF, through increasing the level of NO, is involved in the reduction of ILP in ischemic gerbils, because NO itself may directly inhibit lipid peroxidation by intercepting alkoxyl and peroxyl radical intermediates and thus terminating chain propagation reaction [66], [67]. Di Loreto et al. [18] also proposed that ELF-MF can simultaneously activate pro- and antioxidants. They applied ELF-MF (50 Hz, 0.1 and 1 mT) on cortical neurons and beside increased production of ROS and malondialdehyde (parameter of lipid peroxidation), they also found increased expression of brain-derived neurotrophic factor and nerve growth factor, proteins which participate in free radical clearance [68], [69].

ELF-MF *per se* increased O_2_^−^ content on the 7^th^ exposure day, but when applied in ischemic gerbils it reduced production of this free radical species. Important finding is that the activity of SOD, enzyme which dismutases O_2_^−^, was not increased in ischemic gerbils, but it was increased in ELF-MF exposed gerbils without or with induced global cerebral ischemia on the 7^th^ exposure day. Our findings are in accordance with some papers [16], [53], [60], [70], but also there are some opposite results [17], [71]. This means that ELF-MF activates one of the most important enzyme of antioxidant defense and through reduction of O_2_^−^ level decreases further propagation of oxidative stress event.

The most interesting result of this study is that ELF-MF and ischemia separately increase oxidative stress, but when applied together they have capability to decrease values of measured parameters. One of the possible mechanism(s) underlying the beneficial effects of ELF-MF in the model of global cerebral ischemia could be initial ability of ELF-MF to shift intracellular pH toward more alkaline conditions [36], considering that acidosis is one of the crucial hallmark of ischemic injury [64], which further triggers other neuroprotective pathways. We do not know precisely time profile of evidently common pathways, so it is hard to presume time and place of activation or which pathway dominates and leads to activation of enzymes involved in antioxidant defense. We need additional biochemical and molecular investigations bearing in mind all possible interactions of all elements of central nervous system.
